# Prediction of Crack Width in RC Piles Exposed to Local Corrosion in Chloride Environment

**DOI:** 10.3390/ma16196403

**Published:** 2023-09-26

**Authors:** Wei Shao, Xiaoqing He, Danda Shi, Wenjin Zhu

**Affiliations:** 1 College of Ocean Science and Engineering, Shanghai Maritime University, Shanghai 201306, China; weishao@shmtu.edu.cn (W.S.); ddshi@shmtu.edu.cn (D.S.); 2 Department of Hydraulic Engineering, School of Civil Engineering, Tongji University, Shanghai 200092, China; hexiaoqing0719@163.com; 3 School of Civil and Ocean Engineering, Jiangsu Ocean University, Lianyungang 222005, China

**Keywords:** localized corrosion, corrosion pit radius, steel section loss, crack extension, maximum corrosion level

## Abstract

A novel prediction model for crack development of reinforced concrete (RC) piles with localized chloride corrosion in the marine environment is proposed. A discrete method is used to solve the corrosion pit radius model and a crack extension model is developed to investigate the initiation and extension of cracks. The maximum corrosion degree of the reinforced concrete pile is predicted according to the limit crack criterion, and finally, a sensitivity analysis is carried out on the important parameters of crack extension. The results show that the radius of the corrosion pit, the depth corrosion pit, and the cross-sectional area loss of reinforcement gradually increase as the corrosion level increases. The loss of the local reinforcement section at crack initiation increases with the increase in the ratio of concrete cover to initial diameter and increases with the increase in the pitting factor. The required pit depth for reinforcement cracking increases with the increase in the ratio of concrete cover thickness to diameter. The loss of the cross-sectional area of reinforcement and the radius of the corrosion pit increase with the increase in the initial diameter of reinforcement. Increasing the pitting factor can reduce the pit depth and make the crack width develop faster before reaching the limit crack width. Increasing the concrete cover thickness can provide an improvement in the propagation of cracks. A comparative analysis shows that the localized corrosion pattern is more in conformity with marine engineering practice.

## 1. Introduction

In recent years, the corrosion of reinforced concrete (RC) structures in the marine environment has received great concern. RC structures can be affected by chloride, air, seawater splash, wind, and other external factors during service, which can cause corrosion of reinforced concrete structures. Corrosion can produce some corrosion products on the surface of RC structures, which can cause the volume to increase and create tensile stress around the reinforced concrete. When the maximum tensile strength exceeds the tensile stress of the concrete, the cracking of the concrete cover initiates. Concrete cracking may cause early intrusion of local chloride ions and corrosion of reinforcement. Concrete cracking may cause early localized chloride ion ingress and reinforcement corrosion, corrosion-induced cracks penetrate the concrete cover and provide a way for corrosive media such as chloride ions and oxygen to rapidly enter the reinforcement [1,2]. The cracks start to spread outwards from the steel–concrete interface and finally, lead to the complete cracking of the concrete cover. Concrete cracking leads to changes in the load-bearing performance of RC structures, causing structural instability, and in severe cases, may lead to major accidents. Furthermore, the development of cracks can provide effective information on the corrosion of RC structures, enabling us to monitor the health status of concrete structures in a timely manner. In order to comprehensively predict the durability of reinforced concrete structures under local chloride ion erosion, it is necessary to conduct in-depth research on the mechanisms of corrosion pit development and crack propagation. Therefore, it is very meaningful to study the development of cracks for the durability life prediction and repair of RC structures.

The mechanism of damage and crack expansion in RC structures caused by corrosion and expansion of reinforcement has always been a hot topic of research in this field. In the actual marine environment, chloride generally penetrates into concrete structures in a unidirectional manner, and corrosion may start from the outermost layer of steel bars, causing uneven corrosion on the surface of steel bars [3]. Local invasion of chloride ions causes random corrosion pits of varying shapes and sizes to form on the surface of steel bars. However, the development of cracks is related to the growth of corrosion pits. It is a rare idea to combine the development of corrosion pits with the development of cracks to study the durability of RC piles under local chloride ion erosion.

In recent years, many scholars have studied the changes in the load-bearing performance of RC structures after local corrosion [4,5,6,7,8,9,10]. Only a small number of studies have conducted detailed research on the development of corrosion pits. Kim and Kim [11] estimated the maximum corrosion depth of local corrosion of reinforcement considering chloride ion diffusion. Li et al. [12] conducted tests on pit corrosion of reinforcement in chloride ion corroded concrete and analyzed the relationship between pit depth, residual sectional area, and area of the smaller planar principal moment of inertia and corrosion grade. Darowicki et al. [13] investigated the localized corrosion of stainless steel using optical surface measurement technology and deduced the cumulative distribution function of pitting corrosion. Kioumarsi et al. [14] studied the influence of corrosion pit disturbance on the damage probability of reinforced concrete beams after localized corrosion. These studies have provided us with a better understanding of the generation and development of corrosion pits and also provided a new entry point for the durability research of reinforced concrete structures under localized corrosion conditions.

In addition, some scholars have also conducted research on the cracking and crack development of reinforced concrete. Vidal et al. [15] predicted the crack width of corrosion in reinforced concrete structures and proposed a relationship between reinforcement section loss and cracking. Zhang et al. [16] measured the crack initiation, maximum load, fracture energy, and other parameters of short-cut basalt fiber concrete through tests and studied the crack resistance of concrete. Zhang et al. [17] presented a new model to predict steel corrosion from corrosion cracks based on the average steel section loss parameter. Li et al. [18] proposed a theoretical model for the corrosion crack width of reinforced concrete structures. Zhao et al. [19] presented a concrete cracking model to investigate stress and cracks in concrete cover caused by reinforcing steel corrosion. Shao et al. [20] proposed an analysis model for the durability life of RC piles with chloride local corrosion considering the impact of the crack growth stage on the structure. Guzmán and Gálvez [21] used embedded cohesive cracking finite element analysis to study the effect of localized corrosion on the cracking of concrete cover, deriving different cracking patterns. These studies can help us predict the cracking time of RC structures and have a clear understanding of the development of cracks.

Although the above studies have studied corrosion pits and concrete cracking and crack development, few scholars have combined the growth and crack development of corrosion pits to study the durability life of RC piles. This article aims to combine the growth of corrosion pits with the development of crack width, establish the relationship between the depth and degree of corrosion pits and crack development, and provide new analytical ideas for the development of cracks in locally corroded RC piles. In this paper, a novel prediction model for crack development of RC piles with localized chloride corrosion in the marine environment is proposed. The maximum corrosion degree of the RC pile is predicted according to the limit crack criterion. A discrete method is used to solve the corrosion pit radius model, then crack initiation and extension are investigated based on the crack extension model, and finally, sensitivity analyses are conducted on the important parameters of crack extension.

## 2. Localized Corrosion Modeling

### 2.1. Geometry Model

RC piles exposed to the marine environment are easily corroded by chloride ions. The corrosion of RC structures is generally divided into uniform corrosion and local corrosion, and local corrosion is the key factor for predicting the durability life of RC piles. When reinforced concrete is corroded, corrosion pits are created on the surface of the reinforcement. The location and shape of these corrosion pits are formed randomly, and they are related to factors such as the environment and the level of corrosion. According to existing research, the shapes of corrosion pits may be semi-circular, circular, elliptical, and cup-shaped. In order to facilitate the calculation of the radius of corrosion pits and establish a relationship between local steel section loss and crack width, this paper assumes that the corrosion pits are semi-circular. Considering the uneven corrosion on the surface of the steel bar with a single pit at the top of the steel bar, it is assumed that all corrosion is limited to one pit [22].

### 2.2. Modeling of Corrosion Pits

**Figure 1:**
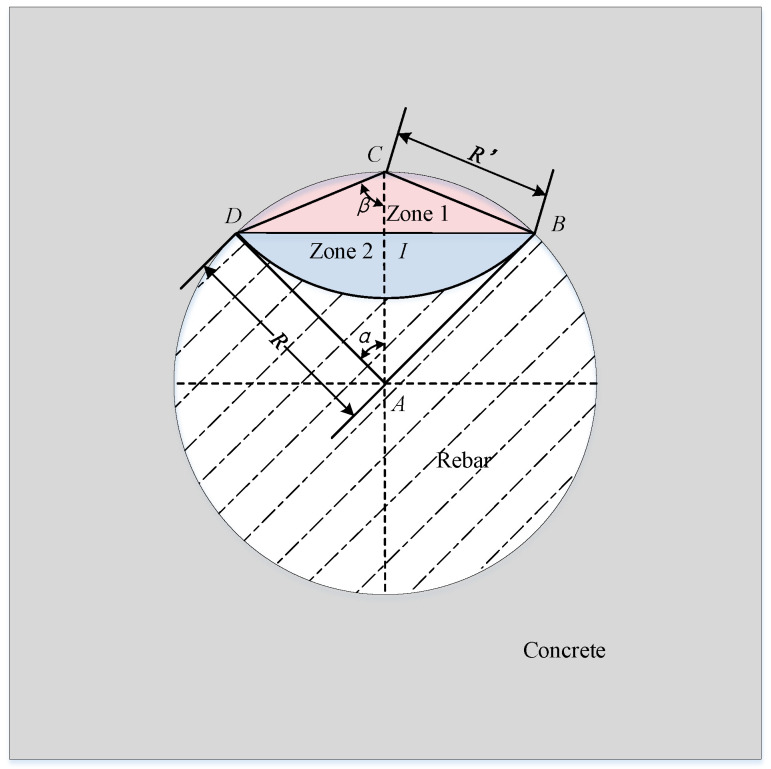
Area loss of reinforcement.

As the corrosion process proceeds, corrosion products can be produced on the surface of the reinforcement, and corrosion products can expand on the surface of the reinforcement. In this paper, the distribution of corrosion pits on the surface of reinforcement is regarded as a simple geometric distribution. As shown in Figure 1, it is assumed that the center *C* of the corrosion crater is directly above the center *A* of the reinforcement. The intersection points of the corrosion pit and reinforcement are *B* and *D*, respectively, the intersection of *AC* and *BD* is *I*, and the radius of the corrosion pit is *R*′. According to Figure 1, the shadow area is the corrosion pit area, which is composed of two semicircles with different radii. The pit area is the sum of the areas of zone 1 and zone 2.

In the corrosion pit model, it is assumed that

(1) IB=ID.

The area of zone 1 can be expressed as

(2) $$A_{1}=A_{S_{ABD}}-A_{T_{ABD}}.$$

The area of triangle *ABD* obtained from geometric knowledge can be expressed as

(3) $$A_{T_{ABD}}=IB\times IA.$$

*IA* can be expressed as

(4) $$IA=\sqrt{OB^{2}-BI^{2}}=\sqrt{R^{2}-R^{\prime }{}^{2}}.$$

Therefore, the area of the triangle *ABD* is expressed as

(5) $$A_{T_{ABD}}=R^{\prime }\times \sqrt{R^{2}-R^{\prime 2}}.$$

The expression for the area of the sector *ABD* can be expressed as

(6) $$A_{S_{ABD}}=\frac{2\pi R^{2}\alpha }{360}.$$

The angle *α* can be expressed as

(7) $$\sin\alpha =\frac{R^{\prime }}{R}.$$

Therefore, the expression for *α* is

(8) $$\alpha =\arcsin\frac{R^{\prime }}{R}.$$

Therefore, the area of sector *ABD* is calculated as

(9) $$A_{S_{ABD}}=\frac{2\pi R^{2}}{360}\cdot \arcsin\frac{R^{\prime }}{R}.$$

In summary, the area of zone 1 is obtained as

(10) $$A_{1}=A_{S_{ABD}}-A_{T_{ABD}}=\left(\frac{2\pi R^{2}}{360}\cdot \arcsin\frac{R\prime }{R}\right)-\left(R\prime \times \sqrt{R^{2}-R^{\prime 2}}\right).$$

Based on the same analysis method, the area of zone 2 can be obtained as

(11) $$A_{2}=A_{S_{CBD}}-A_{T_{CBD}}.$$

*IC* can be expressed as

(12) $$IC=AC-AI=R-\sqrt{R^{2}-R^{\prime 2}}.$$

The relationship between angle *β* and angle *α* is given by

(13) $$\beta =90^{\circ }-\alpha .$$

Therefore, the area of the sector *CBD* is obtained as

(14) $$A_{S_{CBD}}=\frac{2\pi R^{\prime 2}}{360}\left(90^{\circ }-\alpha \right).$$

The area of triangle *CBD* is given by

(15) $$A_{T_{CBD}}=R\prime \times \left(R-\sqrt{R^{2}-R^{\prime 2}}\right).$$

According to Equations (14) and (15), the area of zone 2 is

(16) $$A_{2}=A_{S_{CBD}}-A_{T_{CBD}}=\frac{2\pi R^{\prime 2}}{360}\cdot \left(90-\alpha \right)-R^{\prime }\times \left(R-\sqrt{R^{2}-R^{\prime 2}}\right).$$

Therefore, the corrosion pit area can be expressed as

(17) $$\Delta A_{s}=A_{1}+A_{2}=2\pi \left[\frac{R^{\prime 2}}{360}\cdot \left(90-\alpha \right)+\frac{R^{2}}{360}\cdot \alpha \right]-R^{\prime }\cdot \left[\sqrt{R^{2}-R^{\prime 2}}+\left(R-\sqrt{R^{2}-R^{\prime 2}}\right)\right].$$

Equation (17) can be simplified as

(18) $$\Delta A_{s}=2\pi \left[\frac{R^{\prime 2}}{360}\cdot \left(90^{\circ }-\alpha \right)+\frac{R^{2}}{360}\cdot \alpha \right]-RR^{\prime }.$$

where Δ*A_s_* is the area of the corrosion pit and denotes the loss of cross-sectional area of the reinforcement, *R*′ is the corrosion pit radius and *R* is the initial radius of the reinforcement. In general, the degree of corrosion of reinforced concrete piles is expressed as the percentage mass loss of reinforcement *ρ*, and *ρ* can be calculated as

(19) $$\rho =\frac{M_{1}-M_{2}}{M_{1}}$$

where *M*_1_ and *M*_2_ are the masses of the reinforcement bars before corrosion and the mass after corrosion, respectively. Equation (19) can be rewritten as [23]

(20) $$\rho =\frac{M_{1}-M_{2}}{M_{1}}=\frac{\rho_{rebar}\cdot \Delta A_{s}}{\rho_{rebar}\cdot A_{rebar}}=\frac{\Delta A_{s}}{A_{rebar}}.$$

According to Equations (18) and (20), the following equation can be obtained as

(21) $$\rho A_{rebar}=2\pi \left[\frac{R^{\prime 2}}{360}\left(90^{\circ }-\alpha \right)+\frac{R^{2}}{360}\alpha \right]-RR^{\prime }$$

where *ρ* is the degree of corrosion of the reinforcement, and *A_rebar_* is the initial area of the reinforcement. Equation (21) involves two unknown parameters *ρ* and *R*′, when the degree of corrosion *ρ* is known, the radius of pit radius *R*′ can be obtained for different corrosion degrees. However, since the unknown parameter *R*′ is present in the trigonometric function, the radius of the corrosion pit cannot be solved by simple reduction. Therefore, the discretization method can be used to solve the problem. The corrosion pit radius *R*′ is ranging from 0 to 5. Combined with relevant software programming, when the corrosion degree *ρ* is determined, the corrosion pit radius is discretized into countless points within its feasible range, and the point that makes both sides of the equation infinitely close is found. This point is the optimal solution we are looking for. It is inevitable that using the discrete method to solve the corrosion pit model can result in certain calculation errors *E*, so this article also analyzes the discrete error.

### 2.3. Analysis of Crack Width

After the onset of cracking in RC structures, the corrosion products on the surface can first fill the local pores and then be deposited at the interface between the reinforcement and the concrete. The depth of the corrosion pit required for reinforcement cracking is calculated by [24]

(22) $$x_{0}=7.53+9.32\frac{c}{D_{0}}$$

where *x*_0_ is the corrosion pit depth required for cracking, *c* is the concrete cover thickness (mm) and *D*_0_ is the initial diameter of the reinforcement. The above equation shows that the depth of the corrosion pit required for cracking is related to the ratio of the concrete cover thickness and the initial diameter of the reinforcement. The relationship between the depth of the corrosion crater and reinforcement cross-section loss can be obtained by [15]

(23) $$x=\frac{D_{0}}{\alpha }\left[1-\sqrt{1-\frac{\Delta A_{s}}{A_{rebar}}}\right]\cdot 10^{3}$$

where *α* is the pitting factor, is the ratio of localized corrosion depth to uniform corrosion depth, *α* = 2, for uniform corrosion, 4 < *α* < 8, for localized corrosion, *x* is the depth of the corrosion pit (μm), *D*_0_ is the initial diameter of the reinforcement (mm), *A_rebar_* is the initial area of the reinforcement and Δ*A*_s_ is the loss of reinforcement cross-section (mm^2^). According to Equations (22) and (23), the local steel section loss at the beginning of cracking can be obtained as [17]

(24) $$\Delta A_{s0}=A_{rebar}\left[1-{\left[1-\frac{\alpha }{D_{0}}\left(7.53+9.32\frac{c}{D_{0}}\right)\cdot 10^{-3}\right]}^{2}\right].$$

It should be noted that the local reinforcement section loss at the onset of cracking is determined by Equation (24) without taking into account the properties of the concrete. Based on the experimental data, a linear regression formula obtained by Vidal et al. [15] is used for predicting crack width as

(25) $$w=K\left(\Delta A_{s}-\Delta A_{s0}\right)$$

where Δ*As*_0_ is the loss of the local steel cross-section at the beginning of cracking, and *K* is the slope of the curve (*K* = 0.0575). According to the limit crack criterion, when the crack width exceeds the limit crack value, the reinforced concrete pile loses stability. 

## 3. Analysis Results

**Figure 2:**
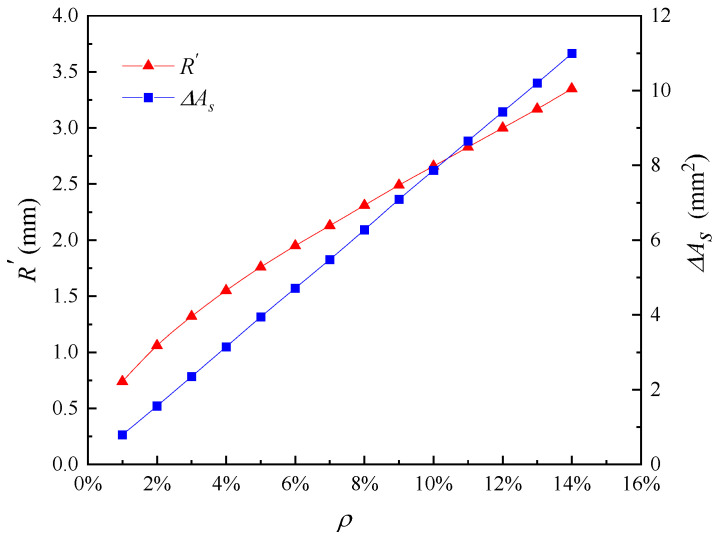
Influence of corrosion degree on radius of corrosion pit and cross-sectional area loss of reinforcement.

**Figure 3:**
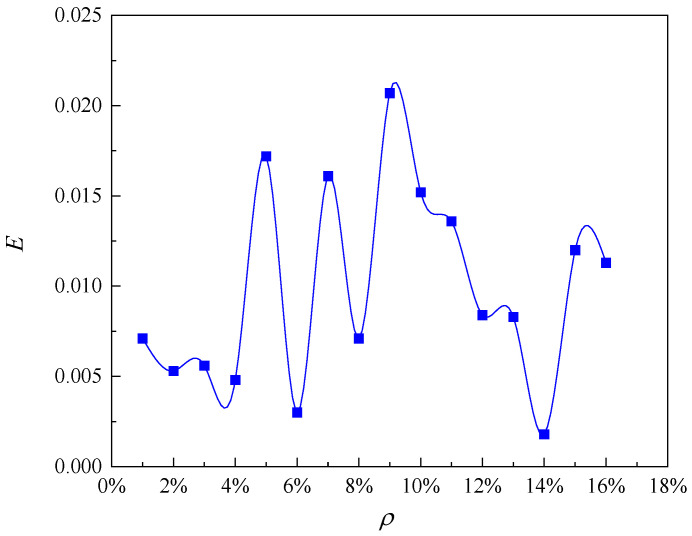
Calculation error of corrosion pit radius.

**Figure 4:**
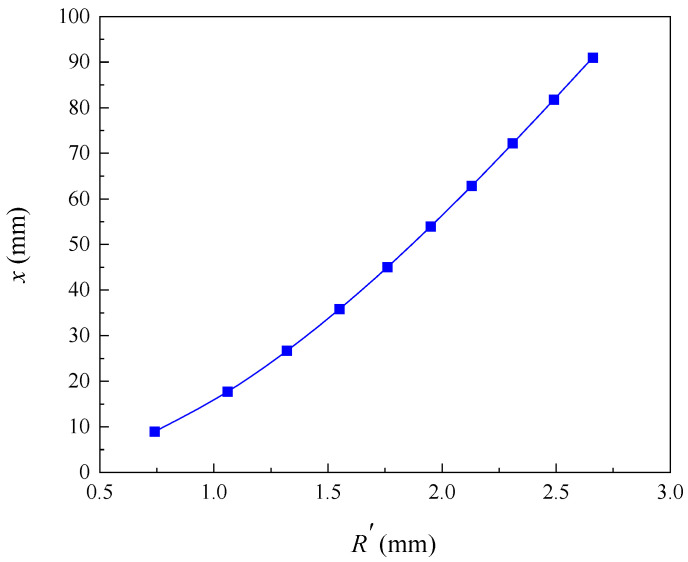
Influence of pit radius variation on corrosion pit depth.

In this paper, the selected limit crack width is *w_lim_* = 0.4 mm. The values of model parameters used in this analysis are shown in Table 1. According to Equation (22), the pit depth at the beginning of reinforcement cracking is 0.054 mm, and according to Equation (24), the local steel section loss at the beginning of cracking is 4.73 mm^2^. The relationship between the radius of the corrosion pit and the loss of reinforcement cross-section with the degree of corrosion is shown in Figure 2. When the corrosion degree increases, the corrosion pit radius gradually increases, and the reinforcement section loss gradually increases. This is consistent with the findings of the experiment by Li et al. [12]. The error analysis caused by solving the corrosion pit radius with the discrete method is shown in Figure 3. The error varies randomly and irregularly as the corrosion level increases from 1% to 16%, while each error is very small; therefore, the influence of the error formed by the discrete method on the results can be ignored. Figure 4 shows the relationship between corrosion crater radius and corrosion crater depth. The depth of the corrosion pit increases with increasing pit radius.

The depth of corrosion pits under the influence of different corrosion degrees and pitting factors is shown in Table 2. When the corrosion degree is 8%, the pit depth decreases from 115.50 mm to 57.75 mm as the pitting factor increases from 4 to 8. The crater depth decreases with the increase in pitting factor. This is because when the corrosion degree is certain, the cross-sectional loss of reinforcement is also a fixed value, and the depth of the corrosion pit is inversely proportional to the pitting factor. When the pitting factor is 6, the pit depth increases from 50.81 mm to 76.99 mm as the corrosion degree increases from 6% to 9%. The corrosion degree increases from 6% to 7%, from 7% to 8%, and from 8% to 9%, and the pit depth increases by 16.53%, 14.69%, and 13.27%, respectively. The depth of the corrosion crater increases slowly. This is because, with the increase in corrosion level, the local corrosion of reinforcement becomes more and more serious. The corrosion products generated by corrosion attach to the surface of reinforcement and block the flow of media required for corrosion such as chlorine ion and oxygen, thus slowing down the development of corrosion to a certain extent.

The crack width under the influence of different corrosion degrees and pitting factors is shown in Table 3. When the corrosion degree is 9%, the crack width reduces from 0.21 mm to 0.03 mm as the pitting factor increases from 4 to 8. The crack width decreases with the increase in pitting factor. When the pitting factor is 5, the crack width increases from 0.03 mm to 0.17 mm as the corrosion degree increases from 6% to 9%. The crack width increases 4.67 times. The crack width increases with the increase in corrosion degree. This is because the progress of corrosion, air, oxygen, and other corrosive media is sufficient, and corrosion can develop rapidly at the beginning. The crack width can expand rapidly before reaching the limit crack width, but after the crack width reaches the threshold, the corrosion rate can slow down because the accumulation of corrosion products restricts the flow of air and so on.

**Figure 5:**
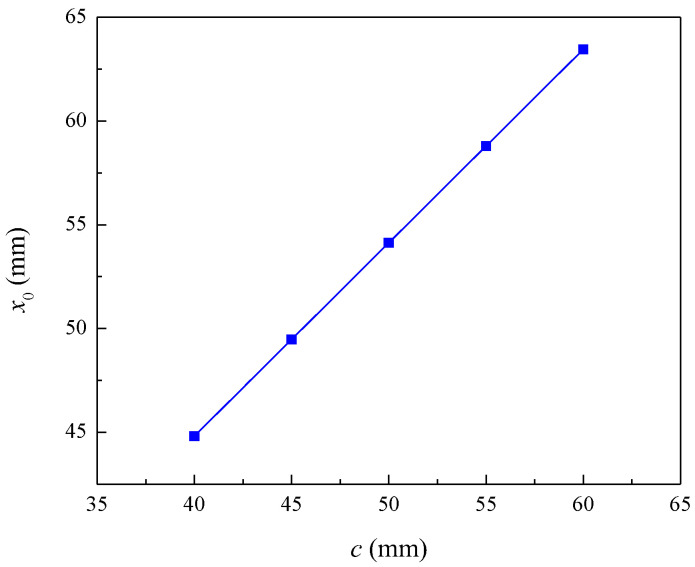
Influence of concrete cover thickness on pit depth during cracking.

The influence of the concrete cover thickness on the required pit depth at the initiation of reinforcement cracking is shown in Figure 5. When concrete cover thickness increases from 40 mm to 60 mm, the corrosion pit depth at the beginning of reinforcement cracking increases from 44.81 mm to 63.45 mm. The depth of the pit required for the initiation of reinforcement cracking increases with the concrete cover thickness. This is because increasing the concrete cover thickness not only increases the diffusion path of the chloride but also decreases the tensile stresses on the outer surface of the concrete cover due to the radial expansion of the corroded reinforcement, thus delaying the initiation of cracking the concrete cover.

The influence of the thickness of concrete cover on crack width is shown in Table 4. When the corrosion degree is 8%, the crack width decreases from 0.14 mm to 0.04 mm as the concrete cover thickness increases from 40 mm to 60 mm. The crack width decreases with the increase in concrete cover thickness. This is because the increase in the thickness of the concrete cover improves the impermeability, corrosion resistance, and other durability of the reinforced concrete, so the crack expansion is improved. Generally speaking, the greater the thickness of the concrete cover, the longer it takes for the external corrosive medium to reach the surface of the steel, the less likely it is for the reinforcement to rust, and the better the durability of the concrete. However, the concrete cover should not be too thick, as the shrinkage and temperature stresses are not well controlled during the hardening process.

**Figure 6:**
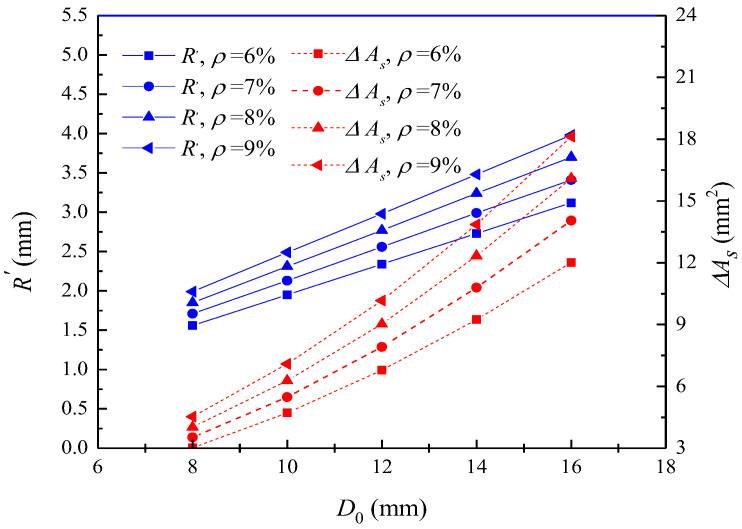
Effect of initial diameter on corrosion pit radius and loss of reinforcement cross-section.

The effect of the initial diameter of reinforcement on the radius of the corrosion pit and the loss of the reinforcement section is shown in Figure 6. When the corrosion degree is 6%, the radius of the corrosion pit increases from 1.56 mm to 3.12 mm with the reinforcement diameter increasing from 8 mm to 16 mm. At the same corrosion degree, the radius of the corrosion pit tends to increase with the increase in the initial diameter of reinforcement. When the initial diameter is 10 mm, the corrosion pit radius increases from 1.95 mm to 2.49 mm as the corrosion level increases from 6% to 9%. At the same initial diameter, the radius of the corrosion pit tends to increase with the increase in corrosion degree. This shows that the corrosion radius and the loss of cross-sectional area of reinforcement increase with the increase in the initial diameter of reinforcement.

**Figure 7:**
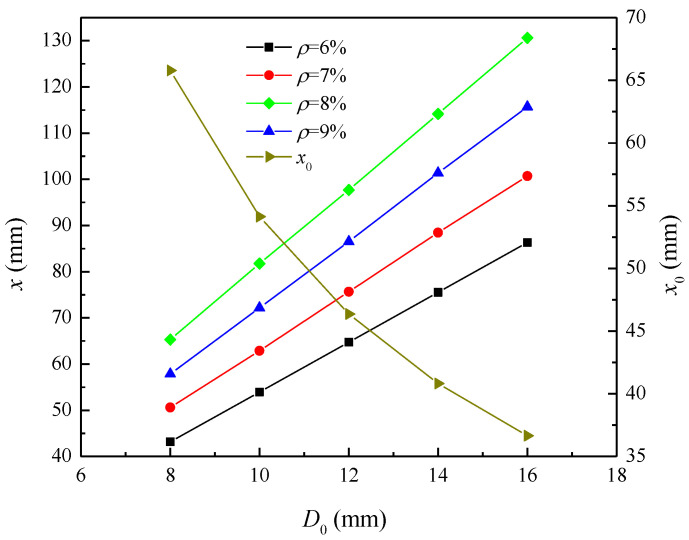
Influence of initial diameter on the pit depth at the initiation of cracking and the pit depth during corrosion.

Figure 7 shows the influence of the initial diameter of reinforcement on the depth of the corrosion pit at the beginning of reinforcement cracking and in the process of corrosion. When the corrosion degree is 6%, 7%, 8%, and 9%, the corrosion pit depth increases from 43.16 mm to 86.32 mm, from 50.61 mm to 100.71 mm, from 57.85 mm to 115.70 mm, and from 65.31 mm to 130.61 mm, as the initial diameter of reinforcement increases from 8 mm to 16 mm. At the same corrosion degree, the pit depth increases with the increase in the initial diameter of the reinforcement. When the initial diameter of the reinforcement increases from 8 mm to 16 mm, the required corrosion pit depth for the cracking of the reinforcement decreases from 65.78 mm to 36.66 mm. The depth of the corrosion pit required for reinforcement cracking reduces as the initial diameter of the reinforcement increases.

**Figure 8:**
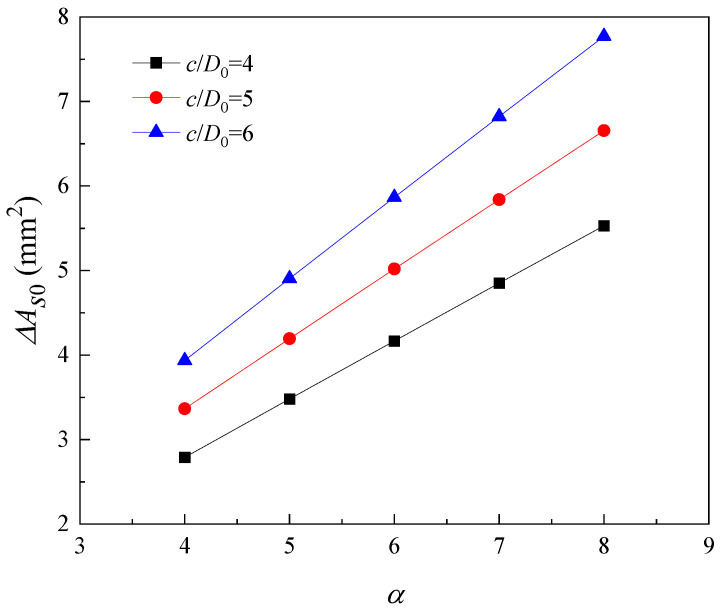
Factors affecting localized steel section loss at crack initiation.

The localized steel section loss at the initiation of rebar cracking is related to the ratio of concrete cover thickness to rebar diameter and the pitting factor. As shown in Figure 8, when the pitting factor is 6, the local steel section loss at the beginning of cracking increases from 4.12 mm^2^ to 5.87 mm^2^ as the ratio of concrete cover to steel diameter increases from 4 to 6. In addition, the local steel section loss at the beginning of cracking increases with the increase in pitting factor. This is because the increase in pitting factor means that the ratio of local corrosion degree to uniform corrosion degree increases, the local corrosion degree increases, and then the loss of cross-sectional area of the reinforcement at the beginning of reinforcement cracking increases.

**Figure 9:**
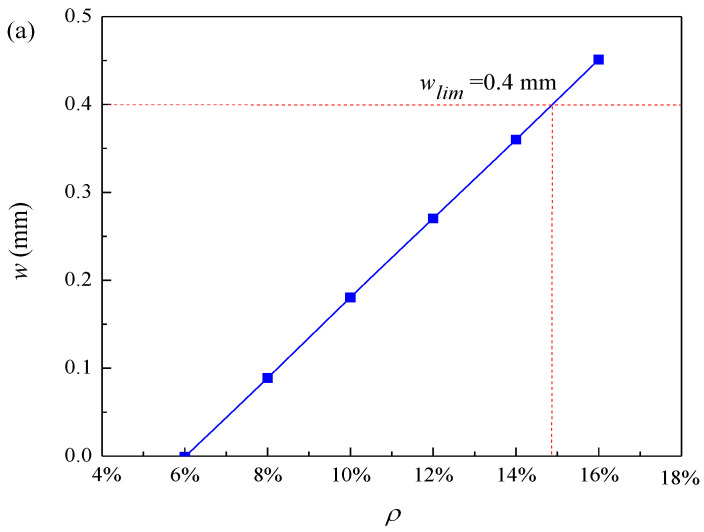

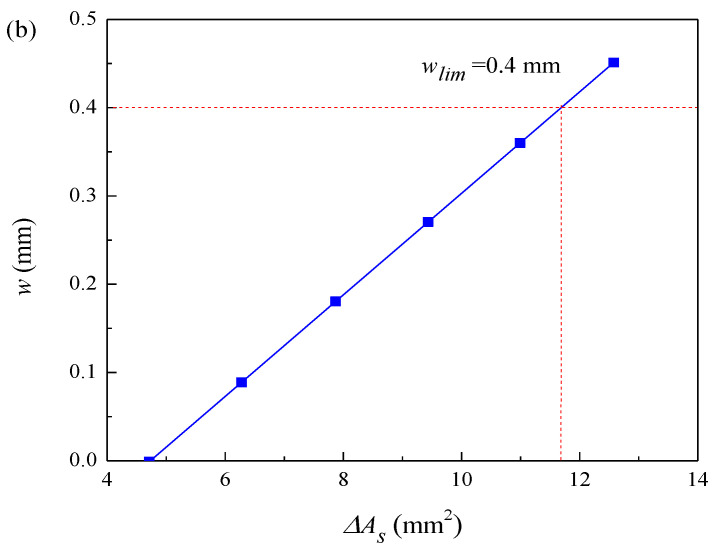
Effect of different factors on crack width: (**a**) corrosion degree and (**b**) loss of reinforcement cross-sectional area.

The influence of corrosion degree on crack width is shown in Figure 9a. When the corrosion level is approximately 15%, the crack width reaches the ultimate crack width of 0.4 mm, and the structure becomes unstable. The influence of the loss of cross-sectional area of reinforcement on the crack width is shown in Figure 9b. When the crack width reaches the limit crack width, the loss of cross-sectional area of reinforcement is about 11.79 mm^2^. The crack width increases with the increase in corrosion degree. This is because the reinforcement corrosion becomes more serious with the increase in corrosion degree. The crack gradually expands with the reinforcement corrosion until the structure is unstable.

**Figure 10:**
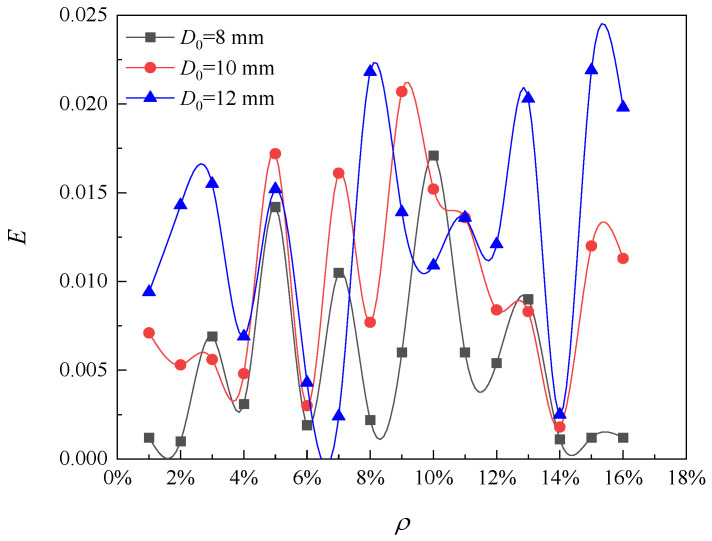
Effect of diameter change on error.

Figure 10 shows the influence of the initial diameter of reinforcement on the model error of the corrosion pit radius. As the corrosion level increases from 1% to 16%, the error curves follow very similar trends for initial steel diameters of 8 mm, 10 mm, and 12 mm. The error varies within the range from 0 to 2%. Therefore, it can be concluded that the initial diameter of reinforcement has little effect on the variation trend of the error of the pit radius model, and the error caused by the solution of the pit radius model can be ignored.

## 4. Discussion

The corrosion of steel bars can generally be divided into two types: uniform corrosion and local corrosion. The former assumes uniform corrosion on the surface of steel bars, uniform expansion stress generated by corrosion, and uniform distribution of corrosion products and depth along the surface of steel bars. The impact of uniform corrosion on piles is mainly proactive damage. The latter focuses on the study of local corrosion on the surface of steel bars, which is defined as local corrosion on the metal surface. It refers to the local dissolution of the metal caused by the rupture of the surface passivation protective film, which is limited to a single point or small area, and ultimately forms a form of voids (i.e., corrosion pits or cracks), which is a passive injury. Under the assumption of uniform corrosion, the research generally takes the time of corrosion initiation or the time of the first crack appearance as the standard for judging the durability of piles. Under the assumption of local corrosion, the time when cracks develop into severe cracks is used as the criterion for judging the instability of concrete piles. Kim and Kim [11] carried out a numerical analysis of localized corrosion (two-dimensional corrosion) of reinforced concrete structures. The results show that the depth of localized corrosion is 3.5 times greater than the depth of uniform corrosion after 3.5 years from the onset of corrosion. The concrete surface cracking time in localized corrosion mode is earlier and the vertical surface displacement is much greater than in uniform corrosion [21]. It has been shown that in the localized corrosion mode, cracks appear earlier on the concrete surface and the pressure to crack the concrete surface is much less than in the uniform corrosion mode. In addition, localized corrosion requires less loss of reinforcement section and shorter periods to reach the limit state of the concrete structure [27].

Table 5 shows the predicted durability life of piles under two different corrosion mode assumptions. It should be noted that the durability life of piles obtained under both assumptions is based on the theoretical model. In order to reduce errors, we ensure that the same parameter values are consistent for both corrosion modes and use the same judgment criteria to predict the durability life of piles. From Table 5, it can be seen that the predicted durability life of piles under uniform corrosion mode is much smaller than that under local corrosion mode. This is because the assumption of uniform corrosion greatly simplifies the modeling process, while in fact, the predicted durability life based on the assumption of uniform corrosion only indicates the beginning of corrosion, and the pile has not yet been adversely affected by potential inhibition of its function and performance. In general, corrosion of steel bars occurs simultaneously with uniform corrosion and local corrosion, but the harm of local corrosion to reinforced concrete structures is much greater than that caused by uniform corrosion. According to the short plate effect, the first cause of instability of RC structures mostly depends on the degree of local corrosion. Local corrosion causes passive damage to RC structures. When RC piles are subjected to local corrosion, the surface of the reinforced concrete expands, and the passivation film on the surface ruptures locally, causing concrete cracking. The effective area of the steel bar surface continues to decrease, the bonding between the steel bar and the concrete protective layer deteriorates, and the strength of the pile structure continues to deteriorate, which may lead to local instability of the pile and a decrease in its integrity and safety.

In conclusion, it is too conservative to use the uniform corrosion model to study the corrosion of reinforced concrete structures, and there is a gap in the actual engineering conditions. The cracking time of the concrete surface, the crack initiation time, and the time when the cracks reach the ultimate crack are much longer in the uniform corrosion model than in the localized corrosion model. Therefore, in order to be more relevant to the actual marine engineering, the crack analysis of reinforced concrete structures in localized corrosion mode should be developed as much as possible. This paper proposes a new method for predicting cracks in concrete piles under localized corrosion of chloride in the marine environment, and the predicted results in this paper are in conformity with many experimental results. However, the shape, size, and spatial distribution of the specific corrosion pits and cracks need to be investigated in more depth. For example, by enhancing the identification technology of corrosion pits, the size and shape of corrosion pits at different positions on the surface of steel bars can be identified, and the impact of corrosion pit growth of different shapes on the durability of piles can be further studied.

## 5. Conclusions

This article is focused on the corrosion pits and their effect on the development of cracks in RC piles. In this paper, a novel prediction model for crack development of RC piles with local chloride corrosion in the marine environment is proposed. The generation and development of cracks in RC piles subjected to localized corrosion by chloride ions are studied and a sensitivity analysis of the important parameters of the cracking phase is carried out. Therefore, the following conclusions can be obtained:

(1) The radius of the corrosion pit, loss of cross-sectional area of reinforcement, and corrosion pit depth increase as the corrosion level increases. The corrosion depth decreases with the increase in pitting factor and increases with the increase in corrosion pit radius and initial diameter of steel bars. The crack width decreases with the increase in pitting factor and protective layer thickness. During the initial period of the crack, the development rate of corrosion pits is relatively fast, but before reaching the limit crack, the development of the corrosion pits becomes slow.
(2) The required pit depth for reinforcement cracking increases with the increase in the ratio of concrete cover thickness to diameter. The loss of the cross-sectional area of reinforcement and the radius of the corrosion pit increase with the increase in the initial diameter of reinforcement. The loss of the local reinforcement section at the beginning of reinforcement cracking increases with the increase in the ratio of concrete cover thickness to initial diameter and increases with the increase in the pitting factor.
(3) According to the limit crack criterion, the maximum corrosion degree of the reinforced concrete pile is about 15%. The error caused by the discrete method can be ignored. The above analysis results are obtained without considering the influence of concrete characteristics on steel section loss.
(4) The prediction of the durability life of chloride ion corroded piles under the assumption of uniform corrosion is too conservative. In fact, the predicted durability life based on the assumption of uniform corrosion only indicates the beginning of corrosion, and the pile has not yet been adversely affected by the potential inhibition of its function and performance. In order to more accurately predict the time when the crack reaches the limit crack, the durability prediction of piles should be carried out under the assumption of local corrosion, rather than uniform corrosion. When evaluating the durability life of RC piles in actual service, engineers should pay more attention to the local corrosion on the surface of RC piles. This paper proposes a new method to predict the cracking of concrete piles by localized corrosion of chloride in the marine environment. However, the impact of different shapes and spatial distributions of corrosion pits on crack width is currently unclear. If advanced corrosion pit identification technology can be combined to study the impact of different shapes and spatial distributions of corrosion pits on the development of crack width, it will provide effective measures for the durability evaluation of future RC structures.

## Figures and Tables

**Table 1:** Values of main parameters used in the analysis.

Parameters	Unit	Values	Description	Reference
*a*	mm	190	Inner radius of pile	-
*b*	mm	300	Outer radius of pile	-
*D*_0_	mm	10	Initial diameter of steel bar	-
*c*	mm	50	Concrete cover thickness	-
*w_lim_*	mm	0.4	Limit crack width	[25]
*α*	-	5.65	Pitting factor	[15]
*K*	-	0.0575	Slope of curve	[26]

**Table 2:** Depth of corrosion pits under different degrees of corrosion and pitting factors (mm).

	*α*	4	5	6	7	8
*ρ*						
6%		76.20	60.97	50.81	43.55	38.10
7%		88.82	71.06	59.21	50.75	44.41
8%		101.96	81.57	67.97	58.26	50.98
9%		115.50	92.40	76.99	66.00	57.75

**Table 3:** Crack width under different degrees of corrosion and pitting factors (mm).

	*α*	4	5	6	7	8
*ρ*						
6%		0.08	0.03	-	-	-
7%		0.12	0.07	0.03	-	-
8%		0.16	0.12	0.07	0.03	-
9%		0.21	0.17	0.12	0.07	0.03

**Table 4:** Crack width under different concrete cover thicknesses and corrosion levels (mm).

	*c*	40	45	50	55	60
*ρ*						
6%		0.05	0.02	-	-	-
7%		0.09	0.07	0.04	0.02	-
8%		0.14	0.11	0.09	0.07	0.04
9%		0.18	0.16	0.14	0.11	0.09

**Table 5:** Prediction results of durability life of piles under different corrosion modes.

Corrosion Mode	Method	Model	Durability Life (Years)
Uniform corrosion [28]	Determination method	*F*(*t*) = *C_th_* − *C*(*r*,*t*)	34.8
	Reliability method	*P_f_*(*t*) = *P*[*F*(*t*) ≤ *0*] = *P*[*C_th_* ≤ *C*(*r*,*t*)], *T_i_* = [*P_f_*(*t*) ≥ *P_fmax_*]	27.4
Localized corrosion [20]	Determination method	*T_sp_*_,*lim*_ * = T_i_ + T_cr_ + T_cp_*	77.60

## Data Availability

The data presented in this study are available on request from the corresponding author.
